# Rapid evidence review: Policy actions for the integration of public health and health care in the United States

**DOI:** 10.3389/fpubh.2023.1098431

**Published:** 2023-03-29

**Authors:** Jennifer S. Lin, Elizabeth M. Webber, Sarah I. Bean, Allea M. Martin, Melinda C. Davies

**Affiliations:** Kaiser Permanente Evidence-based Practice Center, Kaiser Permanente Center for Health Research, Portland, OR, United States

**Keywords:** public health, health care, integration, policy, review

## Abstract

**Objective:**

To identify policy actions that may improve the interface of public health and health care in the United States.

**Methods:**

A rapid review of publicly-available documents informing the integration of public health and health care, and case examples reporting objective measures of success, with abstraction of policy actions, related considerations, and outcomes.

**Results:**

Across 109 documents, there were a number of recurrent themes related to policy actions and considerations to facilitate integration during peace time and during public health emergencies. The themes could be grouped into the need for adequate and dedicated funding; mandates and shared governance for integration; joint leadership that has the authority/ability to mobilize shared assets; adequately staffed and skilled workforces in both sectors with mutual awareness of shared functions; shared health information systems with modernized data and IT capabilities for both data collection and dissemination of information; engagement with multiple stakeholders in the community to be maximally inclusive; and robust communication strategies and training across partners and with the public.

**Conclusion:**

While the evidence does not support a hierarchy of policies on strengthening the interface of public health and health care, recurrent policy themes can inform where to focus efforts.

## 1. Introduction

The importance of coordination between public health (PH) and health care (HC) entities became highly apparent during the COVID-19 pandemic. For most PH jurisdictions, the lack of an integrated epidemic response model impaired the ability of both PH and HC entities to respond in a timely and effective manner to mitigate the high rates of COVID-19 transmission and its subsequent morbidity and mortality. Due in large part to the historical evolution and financing of the two in the United States, the PH and HC sectors lack a structured partnership with one another, which has greatly hindered coordination and innovation around the delivery of shared core functions. While there is abundant literature on the need for and barriers to such partnerships, as well as case studies of integrated models of PH and HC, it is less clear what local, state, and federal policy actions are needed to improve the interface between PH and HC. In support of a planned Delphi Process to develop an actionable policy agenda, Kaiser Permanente commissioned this rapid review of publicly available documents on the integration of PH and HC. *Integration* is an imprecise term and meant, in this report, to encompass the full continuum of coordination from cooperation to collaboration, to partnership, to merger (i.e., single entity).

## 2. Methods

We searched Medline ALL *via* Ovid from 2000 through 18 April 2022 to identify English language published evidence, expert or consensus recommendations, concept papers, and case studies of optimal models on the integration of PH and HC. The search was designed to capture papers on PH integration with HC. The concept of PH was captured with the terms “public health” or “population health” or “community health.” The concept of HC included components of a health systems (e.g., primary care, hospitals, health insurance) and health planning (e.g., delivery of care, health policy trends, practice, or local government). We also included terms to capture settings or instances in which integration or coordination would be necessary (e.g., public health emergencies, disaster planning). We supplemented searches with expert input and reference lists of selected documents.

We communicated in real time with our Office of Community Health liaisons at Kaiser Permanente on refining our inclusion criteria as we reviewed titles and abstracts. We included any document that explicitly focused on the integration or interface between PH and HC. HC included health systems (hospital and ambulatory), health plans, long-term care, pharmacies, laboratory services, inclusive of medical, mental/behavioral, and dental services. We included of countries with different PH and HC infrastructures; however we excluded documents focusing only on low- and middle-income countries. We also conducted targeted searches for white papers or policy documents on PH and HC resilience, data sharing and IT interoperability, and international models of the integration of PH and HC. Targeted searches involved searching keywords in Google, government websites, and the websites of other organizations of interest.

**Figure 1:**
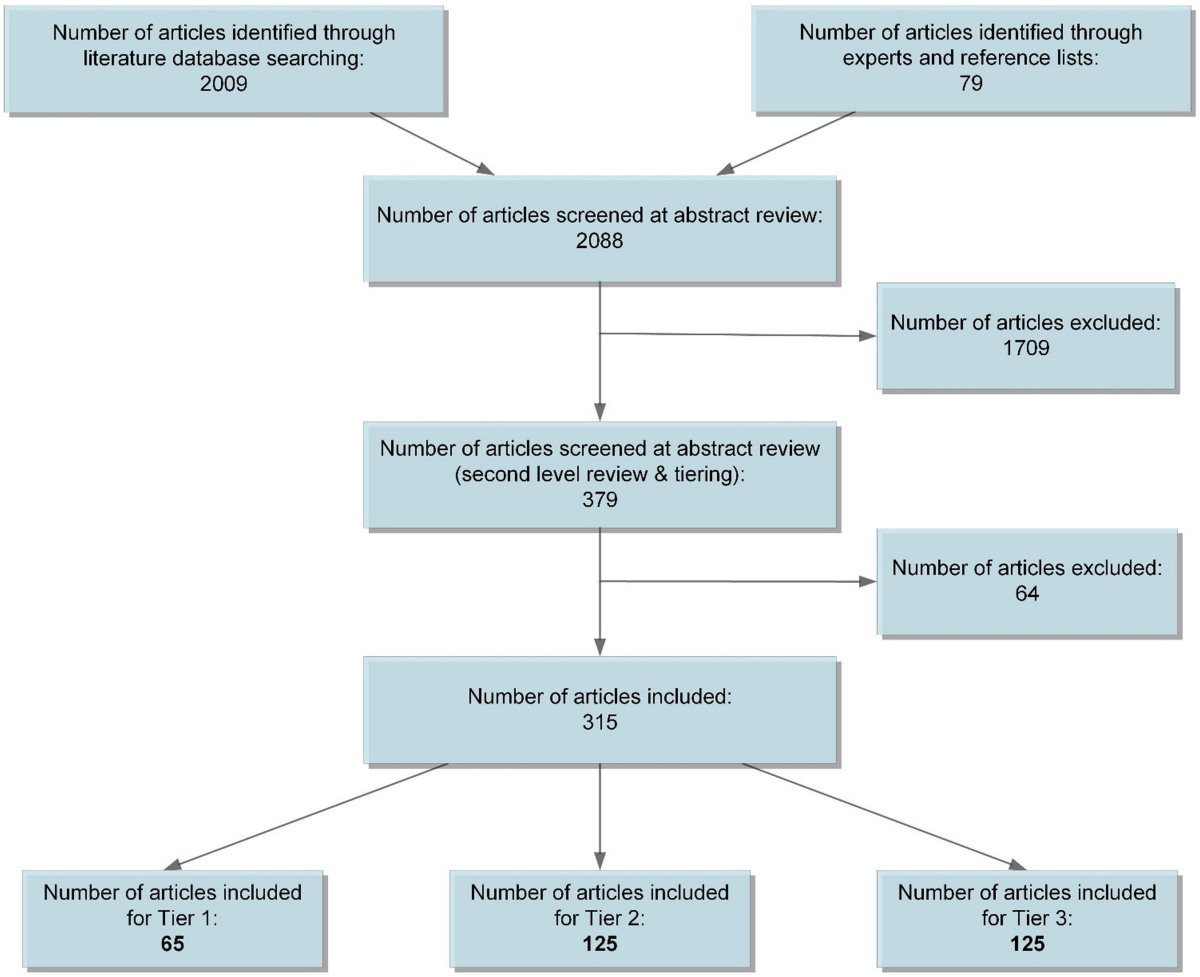
Article flow.

We reviewed titles and abstracts for inclusion. If abstracts were not available, or inclusion could not be determined by title and abstract only, we reviewed the full text for inclusion (Figure 1). After inclusion, we categorized sources into three tiers. Tier 1 consisted of exemplar papers meeting inclusion criteria to be used as our primary source of data (e.g., recent guidance/statements, expert panel or large consensus papers, scoping or systematic reviews, collections of case examples informing broader guidance statements, case studies of multinational efforts). Tier 2 consisted of papers that met inclusion criteria but were single expert or opinion pieces, older publications, and/or individual case studies. Tier 3 consisted of papers that only marginally met our inclusion criteria (i.e., may not have specified collaboration between PH and HC, do not inform policy actions or considerations). Within each tier, we identified two major categories of documents: those generally addressing the integration of PH and HC or primary care and those specific to emergency preparedness and response.

Included articles were not critically appraised for risk of bias. We abstracted main findings focusing on policy actions and considerations from all Tier 1 articles. We then identified shared themes (of actions and considerations) and grouped these themes into general domains. For Tier 1 articles that included an intentional effort to capture a wide representation of case examples (e.g., literature or scoping review), we also recorded when case examples were cited or used to support the policy recommendations. When possible, we added more granularity to policy actions and considerations from these case examples. We then abstracted the main findings from Tier 2 articles and cross-walked these findings with findings from Tier 1 papers to make sure we had achieved saturation of themes. We *a priori* planned only to abstract Tier 3 articles if saturation of content with Tier 2 articles was not achieved. We did not abstract findings from Tier 3 articles. These themes of potential policy actions and considerations are summarized in two main tables (Supplementary Tables 1, 2). We also abstracted general details from Tier 2 case examples of PH and HC integration, noting if objective measures of success were reported. Published studies reporting objective measures of success are summarized in a separate table (Supplementary Table 3).

A draft of this review underwent external peer review by eight experts in public health, population health, community health, emergency preparedness and response, and laboratory services.

## 3. Results

### 3.1. Categorization of included studies and their policy themes

**Figure 2:**
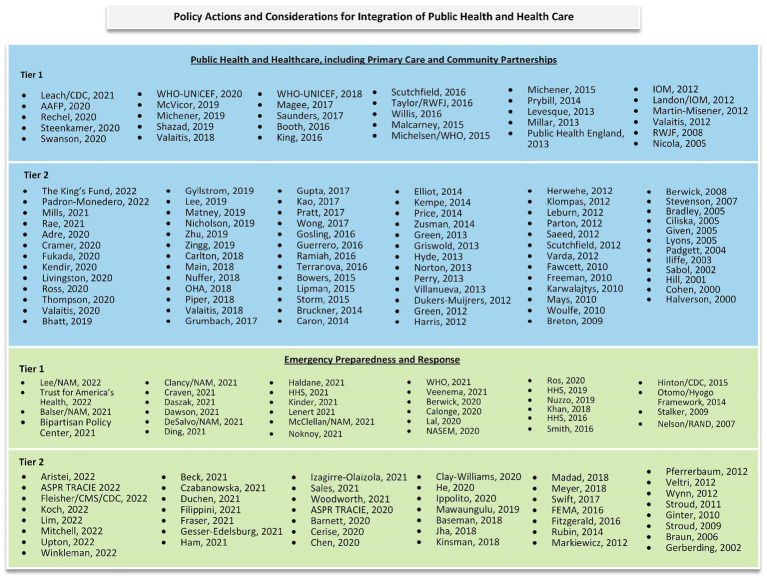
Landscape of included evidence.

For this review we included 109 Tier 1 and Tier 2 documents addressing the integration of PH and HC (including PC) (1–109) and 76 on integration in the context of emergency preparedness and response (Figure 2) (110–185). Many of the articles that reported on the integration of PH and HC were on multisectoral partnerships or collaborations, of which PH and HC were part. Documents focused on bolstering the infrastructure or resilience of PH, without explicit mention of actions or considerations around the integration of PH and HC, were not included (186–190).

Within in each body of literature, we grouped policy actions and considerations for the integration of PH and HC into eight domains: (1) Funding and finance, (2) Governance and legal, (3) Alignment of the core functions that overlap between PH and HC (i.e., surveillance of population health [systematic and opportunistic], health protection and disease control [communicable and non-communicable], health promotion and action on upstream drivers of health, prevention of disease and injuries, and health advocacy), (4) Quality improvement, (5) Physical infrastructure, medical supplies, technologies, and supply chains, (6) Leadership and workforce development, (7) Data and IT capabilities, and (8) Multisectoral partnerships and public engagement. These domains are not mutually exclusive and in some cases the actions and considerations listed apply to multiple categories.

In most instances, policy actions and considerations were stated without specific mention of who should enact or consider these policies (i.e., which entities, organizations, governing bodies). Likewise, in some instances, the actions and considerations could be enacted or considered at multiple levels (e.g., local, regional, state, or national levels), but this was also, most often, not specified.

Although many documents were supported by explicit efforts to identify case examples, often it was not clear to what extent findings or recommendations were supported by ‘successful’ case examples. In addition, definitions or objective metrics of success were not commonly detailed in these reports. Many case examples demonstrate proof of concept and/or subjective assessment of partners’ satisfaction. When documents were explicit that case examples supported their conclusions or recommendations, statements were general in nature (e.g., most of the successful collaborations included pre-existing partnerships).

### 3.2. Public health and health care (including primary care)

Most of the documents addressing the integration of PH and HC focused specifically on primary care (PC) or multisectoral partnerships of PH, HC, and community. Articles covered a wide range of conditions and topics including maternal and child health, chronic diseases (e.g., diabetes, cardiovascular disease), communicable diseases (e.g., HIV and sexually transmitted infections, tuberculosis), cancer, behavioral risk factors (e.g., tobacco use), oral health, multimorbidity; models of care and care settings (e.g., federally qualified health centers, palliative care, long-term care, pharmacy); populations (e.g., pediatric, Medicaid); and provider type (e.g., nurses). While some of these documents focus narrowly on the overlap or integration of PH and HC sectors to deliver individual health care, many more address greater alignment of essential public health services that go beyond public health clinics or publicly funded HC.

Common policy actions and considerations articulated in the documents primary clustered around the need for (Supplementary Table 1):

- Adequate financial resources to improve population health (e.g., adequate base funding, long-term funding, funding to maintain data systems), dedicated funding for collaboration (personnel/programs, training);
- Different payment models (i.e., value-based payment and move away from fee-for-service payment) and purchasing and payment systems to foster reorientation in models of care that integrate PH and PC, or offer adequate remuneration of proactive population health activities and services;
- Mandates for PH and HC (including mental health) integration;
- Shared governance structure (or entities) with defined charter and strategic plan to improve population health, developed with all relevant partners (which may go beyond PH and HC);
- Implementation of models of care that promote high-quality, people-centered, PH and PC integrated health services throughout the life course;
- Application of population health approach and focus on upstream drivers of health (e.g., social determinants of health);
- Robust communication channels between different entities in PH and HC (e.g., timely, multilayered, transparent);
- Infrastructure with equitable distribution of telehealth and health data systems across care settings, sectors and geographic locations;
- Learning environment that integrates quality improvement for patient care and program planning, and accountability to stakeholders;
- Shared or aligned multilevel and distributed leadership;
- Skilled workforce with training for a new set of core competencies (e.g., partnering and team-based care, quality improvement, IT skills, and communication skills);
- Modernized multisectoral data system, with oversight and data linkage by personal or regional identifier, as well as linkage between health and socioeconomic information to address equity; and
- Community engagement with a broad range of stakeholders from private and public sectors with adequate bidirectional communication channels, leveraging digital technologies.

Case examples illustrate that integration of PH and PC may be at the level of delivery of care (e.g., community health clinics in the United States) or integrating PH functions into PC such that they function as one entity (e.g., geographically based integrated care systems in the United Kingdom). Case examples with objective metrics of success demonstrate that the integration of PH and HC in the form of maternal child programs, communicable disease prevention and control programs (e.g., HIV), health information exchanges, health promotion and health protection programs, chronic disease prevention and management, as well as efforts focusing on youth health, women’s health, mental health and working with vulnerable populations, can improve access to care and patient health outcomes (Supplementary Table 3).

### 3.3. Emergency preparedness and response

Most of the emergency preparedness documents were in response to infectious disease PH emergencies (e.g., COVID-19, H1N1) or intended to be broadly inclusive of any PH emergency. A handful of articles focused on other threats, specific topics, or certain populations (e.g., bioterrorism, IT specific, pediatric populations). Common policy actions and considerations articulated in the documents primary clustered around the need for (Supplementary Table 2):

- An increase in flexible and sustained (and stable) funding (personnel/programs, training, infrastructure, data systems);
- Mandates and incentives for PH and HC integration;
- Formal governance structures (or entities) for integration assigning clear roles and responsibilities for each partner;
- Alignment of delivery of shared core functions of PH and HC (e.g., for emergency and non-emergency health services, health risk assessment and surveillance);
- Physical infrastructure that can support surge capacity and weather interruptions, and that leverages telehealth for delivery of core functions;
- Robust measurement strategy to support emergency response, as well as metrics and accountability for performance (to include health equity);
- Dedicated joint leadership between PH and HC with a clear command structure;
- Skilled workforce with adequate (flexible) capacity, training, and support (for mental health and retention);
- Modernized shared data system across sectors (including social sectors) and routinely disaggregated data by key social risk factors (including race/ethnicity); and
- Investment in community partnerships and public engagement with adequate, bidirectional channels of communication that can be a trusted source of timely information as well as used to obtain feedback from public.

Although published case examples of PH and HC integration in the setting of public health emergencies with objective metrics of success are limited, examples demonstrate that coordination of delivery of care using multisectoral coordination or partnership (e.g., PH, hospital, PC, pharmacies, community) can offload hospital demand, improve access to care, and improve vaccination rates during infectious disease outbreaks (Supplementary Table 3).

## 4. Discussion

This rapid review identified a large volume of published or public documents that can inform policy actions and considerations to support the integration of PH and HC in general or specific to PH emergencies. Although these documents span over 20 years, the general themes are quite consistent over time. Because the path of least resistance will always be to work unilaterally, a deliberate and concerted effort must be made to effectively integrate PH and HC. Dedicated funding, incentives, and governance in a hospitable financial and legal environment, as well as a concerted effort by leadership across the federal, regional/state, and local levels is essential for successful integration. This integration extends to multiple entities within PH and HC that are less often acknowledged (e.g., laboratories, mental health providers, pharmacies, long-term care facilities) but, as demonstrated by the COVID-19 pandemic, are critical elements to PH and HC. Ultimately, integration of PH and HC will allow for a proactive rather than reactive management of individual and population health, both during ‘peace time’ and during public health emergencies. Addressing population health will necessarily require addressing health equity as well as upstream and social drivers of health.

Integration between PH and HC necessarily evolves over time and represents a continuum from mutual awareness to true partnership (or functioning as a single entity, in some other countries). The eventuality of true partnership in part depends on time, such that the most efficient policies may be those that foster initial phases of integration. Likewise, the most efficient policies may be those that leverage existing collaborations (e.g., healthcare coalitions). Demonstrating return on investment is critical, but it is important to understand that realizing ‘success’ in this endeavor will happen on a time scale different than most health-related innovations and will require development of adequate metrics of success.

In our review, we found that successful PH initiatives with collaborations at the local and regional level were frequently made possible by commensurate funding and policies at the federal level. External events with funding (e.g., state federal tobacco settlement funds, federal emergency preparedness grants and funding) were necessary catalysts for collaboration. Case examples revealed multiple factors influencing successful integration between PH and HC, including government involvement, geographic proximity of partners, shared goal (of population health) with clear roles/responsibilities and shared protocols, aligned leadership, accountability, workforce education and training, and sharing and collaborative use of data and analysis. Since a community is the immediate and ultimate ‘caretaker’ of populations served by PH and HC, communities need to be included in these integrative efforts. While PH and HC may be the core partners, engaging a broad range of private and public community partners (e.g., social services, education, business, faith-based organizations, community-based organizations) will be necessary to respond to public health emergencies and achieve population health goals.

### 4.1. Limitations

This review included only public or published documents since 2000. We acknowledge that the discussion on the integration of PH and HC reaches back further, with much of the seminal work in the 1990s (e.g., Medicine and Public Health, Lasker and Committee on Medicine and Public Health, 1997 191). Although we conducted targeted efforts using experts, selected reference lists, and web searches to supplement database searching, this review is not exhaustive. Additionally, our identification of examples of the integration of PH and HC surfaced successful efforts, rather than analyses of failed PH and HC integration efforts which are also important in developing policy considerations. Nonetheless, we do believe, given the saturation of themes achieved, that this review represents a summary of major policy actions and considerations on the integration of PH and HC.

Due to the rapid nature of this review, there are several important areas related to the integration of PH and HC that are mentioned, but are not comprehensively covered in this report. These areas include: bolstering PH and PH resilience post-pandemic; bolstering under-resourced aspects of HC (i.e., PC, long-term care, safety net care, rural care); bolstering the PH and HC supply chain; implementing population health in HC or PC; hospital or workplace infection prevention and control; addressing social drivers of health and other upstream drivers of health in PH or HC; the current funding and regulatory environment of PH and HC; general data and IT coordination considerations (e.g., technical interoperability); and the role of research and research partnerships (with PH and HC). Additionally, this review includes documents referring to models of care from various countries that integrate or facilitate the integration of PH and PC. While these exemplar models of care are listed, a deep dive on these models of care was not possible. Last, some of the themes for policy actions and considerations relate to health equity (including the equitable distribution of resources), and when explicitly mentioned by source documents this has been documented. However, and especially with current knowledge and context, it can be argued that most if not all the policy actions and considerations should be seen through a health equity lens.

### 4.2. Conclusion

While the evidence does not support a hierarchy of policy actions and considerations (i.e., it is unclear from case examples of successful integration efforts or models of care what components or external factors contributed to their success), commonalities across examples can help inform where to focus. This review suggests that policy actions and considerations to effectively integrate PH and HC during peace time and during public health emergencies include: the need for adequate and dedicated funding for integration, mandates for integration, shared governance for integration, joint leadership that has the authority/ability to mobilize shared assets, adequately staffed and skilled workforces in both sectors with awareness of shared functions, shared health information systems with modernized data and IT capabilities, engagement with multiple stakeholders in the community to be maximally inclusive, and robust communication strategies and training across partners and with the public.

## Author contributions

JL obtained funding, served as the lead on the synthesis of findings, and drafted the manuscript. EW assisted with the synthesis of findings and review of manuscript, as well as conducted the identification of and abstraction from included studies. SB and AM conducted the identification of and abstraction from included studies, as well as created tables and figures. MD conducted all database searches and reviews. All authors contributed to the article and approved the submitted version.

## Funding

Funding for this review was provided by Kaiser Permanente’s Office of Community Health.

## Conflict of interest

The authors declare that the research was conducted in the absence of any commercial or financial relationships that could be construed as a potential conflict of interest.

## Publisher’s note

All claims expressed in this article are solely those of the authors and do not necessarily represent those of their affiliated organizations, or those of the publisher, the editors and the reviewers. Any product that may be evaluated in this article, or claim that may be made by its manufacturer, is not guaranteed or endorsed by the publisher.
