# Supplementary material for: Rapid evidence review: Policy actions for the integration of public health and health care in the United States
Source: Front Public Health. 2023 Mar 29;11:1098431. doi: 10.3389/fpubh.2023.1098431 (PMC10090415; doi:10.3389/fpubh.2023.1098431)
Supplement: Supplementary file 1 [file Data_Sheet_1.docx]

**Supplement**

**Supplement Table 1: Policy actions and considerations for the integration of public health and health care (including primary care),** (note: bolded text signifies action/consideration from document supported by case-examples)

| **Domain** | **Policy Actions and Considerations** |
| --- | --- |
| Funding and Finance | 1. Invest financial resources to initiate and maintain PH and HC/PC collaboration 2. Ensure adequate base funding (minimum necessary funding) for collaborative efforts to improve population-level outcomes, large- and long enough (e.g., 5 years) to change conditions to achieve population-level changes 3. Define cost of partnership, including the infrastructure as well as human resources for working on collaboration 4. Ensure specialized funding for staffing and infrastructure to support multisector partnerships 5. **Broaden and diversify funding sources;^61^** diversify PH financing beyond state and federal funding 6. With declining overall federal, state, and local PH funding, need diversified funding from philanthropies, insurance reimbursement, innovated approaches tapping targeted taxation, private funding, and social impact bonds 7. HC can invest financially in PH infrastructure to support stronger connections between the two, which provides more sustainable funding stream for PH programs 8. **Can be a direct payment from HC to state or county PH department to support staffing or specific program^147^** 9. Coordinated care organizations (CCOs) share financial resources with PH and non-clinical providers and HC providers 10. Add flexibility to PH funding to allow for ease of collaboration between public and private sectors 11. Note: PH funding is primarily through governmental dollars that are restricted for specific purposes and does not lend itself to cross-sectoral partnerships 12. Create distinct pool of funds jointly through PH and HC that support collaborative projects (for common programs, services, or initiatives) 13. **Develop proposals and applications, as well as implement joint grant-funded projects**[**^48,^**](bookmark://_ENREF_48) [**^61^**](bookmark://_ENREF_61) 14. **Ensure one or more “anchor institutions” with dedication to the partnership’s mission and strong commitment to provide ongoing financial support for the partnership**[**^48,^**](bookmark://_ENREF_48) [**^61^**](bookmark://_ENREF_61) 15. Explore professional payment models for physicians that incentivize collaborative efforts 16. Offer avenues for salaried PH and PC physicians for collaborative projects (UK)      1. Explore and implement purchasing and payment systems that foster a reorientation in models of care for population health (which includes the delivery of integrated health services with PC and PH at the core) and that create incentives to improve access, quality, equity and efficiency of care (see Alignment of Core functions) 2. Support continuous improvement of purchasing and payment systems through regular monitoring of incentives and possibly adjustments to the payment method 3. Structure reimbursement systems to pay for evidence-based programs 4. **Develop new financial arrangements (value-based payment model) in co-creation between contracting parties^11^** 5. Incentivize improvement in outcomes rather than delivery of services 6. Improve existing fee-for-service (FFS) payment models to encourage HC engagement in population health, improving RVU remuneration of services that are proactive or focused on barriers of care      1. Examine how to best use the Affordable Care Act’s community benefits requirement for nonprofit hospitals by coordinating the alignment of the data collection process and pooling resources and determining how these can be used to advance and provide funding for public health      1. Provide adequate financing for underfunded sectors, namely PH and PC 2. Note: actions and considerations directed solely at bolstering public health funding are noted in Appendix E 3. Increase investment in prevention 4. Mobilize and allocate funding in ways that promote health equity 5. Work for adjustments in payment models (e.g., advanced PC) and increases in PC spending to facilitate integration work at the practice and community levels 6. Ensure long-term financial support for population health, ideally regarding PH and PC as one health system      1. Demonstrate return on investment of integration efforts 2. Expand research to show return on investment of integration (see Quality Improvement)   Note: impact of changes may take many years, many confounding variables interfere with definitive proof of impact, variability of implementation of integration and current actuarial and fiscal approaches in US do not provide means of appropriately estimating impact, and investment in one sector may have impact on another sector (aka wrong pocket problem)   1. **Develop and disseminate “impact statements” that present an evidence-based picture of the effects the partnership’s efforts are having in relation to the direct and indirect costs the partnership is incurring^61^** 2. Promote transparency by releasing data on budgets and expenditures to the maximum extent      1. Provide funding for improved data and information systems to support accountability and quality improvement (see Data and IT Capabilities) 2. Need financial investment in data infrastructure (not equal across countries, geographic regions, and sectors) and data collection, and to ensure data quality (EU)      1. Ensure public health as well as private/HC laboratories are able to bill insurers for services 2. Bill at competitive rates by providing ongoing direct financing for core PH function and testing related to uninsured populations 3. Require PH laboratory inclusion in networks offered by public and private insurers, including Medicaid managed care organizations, health plans participating in Children’s Health Insurance Program (CHIP), health plans sold in individual and small group markets (inside and outside health insurance exchanges) 4. Network inclusion for all tests or those tests that are related to key conditions, i.e., STI, communicable disease outbreaks, and other health conditions of public health importance |
| Governance and Legal | 1. Establish clear mandates and policies for collaboration 2. Note: **mandates should align with of governmental agenda^81^** and include standards regarding collaboration 3. Local, state, and federal agencies with responsibilities related to population health improvement and hospital and public health associations should adopt policy positions that promote the development of collaborative partnerships involving , public health departments, and other stakeholders focused on assessing and improving the health of the comm hospitals unities they serve 4. Mandate should include health centers document collaboration with their state and/or local health department 5. Purchasers can leverage managed care contracts to compel health plans to formally coordinate with PH on prevention and health promotion 6. PH can set best practices and minimum requirements related to the nature and substance of such coordination agreements 7. Set eligibility criteria for state grant funding opportunities to include requirements that PH and HC providers jointly apply 8. The Substance Abuse and Mental Health Services Administration should encourage state mental health and substance use disorder agencies and other grantees to collaborate with state, local, and tribal public health entities in achieving PH3.0 goals      1. Establish shared governance and focus (vision, mission, and goals) in support of PH and HC partnerships 2. Note: governance and policy frameworks should build partnerships within and across sectors (i.e., transcend silos) and promote community leadership and mutual accountability 3. **Develop clearly articulated shared mandates, goals, and strategic vision**[**^24,^**](bookmark://_ENREF_24) [**^77^**](bookmark://_ENREF_77)**^,^** [**^81^**](bookmark://_ENREF_81) 4. **Systematically communicate shared mandates, goals, and vision to all levels from executive directors to senior managers to front-line practitioners^77^** 5. **Shared mission statement should center on improving health of community^48^** 6. **Focus on improving community health with strategic mission that focuses on linking funding, workforce, technology and/or services across systems^39^** 7. **Goals should incorporate triple aim:^97^ improve the health of populations and reduce inequities, improve patient experience and quality of HC, and reduce healthcare costs; as well as a fourth aim**[**^8^**](http://0.0.0.8/) **improve the provider experience (inclusive of all providers/professionals delivering care)** 8. **Develop goals in partnership with community partners (maintain honesty, consider the different perspectives of partners in setting goals)^96^** 9. **Formal supra- or overarching governance that crosses HC systems and sectors creates opportunities for traditionally siloed functions to develop shared goals, consider comprehensive intervention strategies, and identify complementary services^39^**      1. **Develop strategic plan for coordination or integration in partnership with all relevant stakeholders**[**^48,^**](bookmark://_ENREF_48) [**^61^**](bookmark://_ENREF_61) (see Multisectoral Partnership) 2. **Contractual agreements between jurisdictions and organizations should articulate clear roles and responsibilities^81^** 3. Involve stakeholders early in strategic planning 4. **Planning process should build buy-in and credibility, be on neutral ground, and not be driven by or dominated by any one entity (e.g., state health department)**^96^ 5. State health agencies should convene (or lead this convening of) joint planning efforts that bring voices and assets of each group, including Medicaid, PC, and other key stakeholders 6. **Develop and maintain organizational structures that enable collaboration^61^** 7. **Have a designated body with a clearly defined charter that is empowered by the principal partners to set policy and provide strategic leadership for the partners^61^** 8. **Include cross-system representation on boards and executive committees**[**^39,^**](bookmark://_ENREF_39) [**^47^**](bookmark://_ENREF_47)**^,^** [**^61^**](bookmark://_ENREF_61) 9. **Create intersectoral standing committees with oversight responsibility for their organization’s engagement in examining community health needs, establishing priorities, and developing strategies for addressing them, including multisector collaboration focused on improving community health^61^** 10. Major national organizations representing health and HC should collaborate to foster duplication of these efforts in local communities through a variety of mechanisms 11. Ensure a durable structure is in place to carry out the mission and goals of the collaborative arrangement 12. **Can take the form of a legal entity (tax-exempt corporation that is sponsored by but distinct from sponsoring organizations), formal written affiliation agreement among partners, memorandum of understanding among partners, or other less formal arrangements such as community coalitions^61^ (this last one being the most common in the US)**      1. Coordinate formal agreements and mechanisms to enact them 2. **PH and HC can enter into formal or informal written agreements to coordinate efforts^47^** 3. Define common agenda and the processes and practices needed to execute agenda (e.g., roles, responsibilities, tasks, deliverable to execute, monitor, and evaluate) 4. Clearly state and widely communicate goals and objectives, roles, and responsibilities 5. Define coordinated clinical and administrative services 6. **Organize accountability processes across organization^11^** 7. **Manage competing accountabilities^11^**      1. **Mandate uptake of interoperable electronic health record (EHR) systems (for HC and PH clinics)^5^**      1. Change legislative environment to overcome limitations of data protection without compromising citizen privacy (look to Nordic countries for models of this legislation, e.g., Sweden) to allow for data linkages (EU) |
| Alignment of Core Functions (that overlap between PH and PC) | 1. **Develop and apply models of care that foster and support integration of PH and PC**[**^13,^**](bookmark://_ENREF_13) [**^39^**](bookmark://_ENREF_39)**^,^** [**^72^**](bookmark://_ENREF_72)**^,^** [**^85^**](bookmark://_ENREF_85) 2. **Note: models of care should be suited to the country and local contexts and promote high-quality, people-centered PC and essential PH functions as the core of integrated health services throughout the course of life**[**^13,^**](bookmark://_ENREF_13) [**^72^**](bookmark://_ENREF_72)**^,^** [**^85^**](bookmark://_ENREF_85) 3. **Models of care can include PH professionals integrated into PC, PH services and PC providers working together, comprehensive and proactive benefits packages that include PH, PC services within PH settings, building PH incentives in PC, and multidisciplinary training of PC staff in PH**[**^13,^**](bookmark://_ENREF_13) [**^72^**](bookmark://_ENREF_72) 4. **Examples of models of care include:**[**^13,^**](bookmark://_ENREF_13) [**^72^**](bookmark://_ENREF_72) **integrating PH and PC in community-based care (PC provision integrates PH perspective to meet needs of community)- Community Health Centers (North America), Community-Oriented Primary Care (UK); integrating PC into overall service provision as part of broader health system (integration of PH and PC is embedded in the provision of services that go beyond PC)- Health and Social Services Centers (Quebec, Canada), Accountable Care Organizations (US), Medicare Local (Australia); integrating PH into medical practice through collaborative team work (PH and PH collaborate as teams to improve core functions)- Family Health Teams (Canada), Multidisciplinary Health Clinics (France), Patient-Centered Medical Home (US), General Practitioners with a Special Interest (UK), Primary Health Networks (Australia), General Practice clusters (Hungary)** 5. Collaborative approaches should include program and service delivery with a comprehensive array of services provided by interprofessional teams of service providers 6. Consider adopting new and appropriate health technologies that can facilitate models of care 7. State health agencies and Medicaid offices can increase understanding of state’s Medicaid service delivery models and identify optimum statewide approaches to improve health outcomes and lower cost 8. State health agencies can work with Medicaid offices to develop, pilot, and spread care models linking PH, PC, and community resources      1. **Evaluate models of care that foster and support integration of PH and PC^85^** (see Quality Improvement) 2. Develop and strengthen IT systems for monitoring and facilitating evaluation of models of care (see Data and IT Capabilities) 3. **Support evaluation of existing models of care and work with AHRQ’s Action Networks for diffusion of best practices related to integration of PH and PC (aimed at Centers for Disease Control & Prevention (CDC)/Health Resources and Services Administration (HRSA) but broadly applicable)^85^** 4. **Convene stakeholders at national and regional levels to share best practices in integration of PH and PC (aimed at CDC/HRSA but broadly applicable)^85^**      1. Coordinate delivery of PH and PC services 2. **Develop and maintain collaborative multisystem care pathways that align with identified needs through stages in the patient journey (i.e., patient movements within and between systems) and activities, programs, or services that coordinate activity across the care continuum^39^** 3. **Process changes aimed at streamlining patient navigation across systems and services^39^** 4. Redefine population based on the PH definition as geographic, as opposed to a practice patient panel 5. Consider geographic proximity of integrating programs or services between PH and PC  - **Establish ‘one-stop’ shop centers, where clinical and community-based professionals are brought together at one site (co-location), organized around the needs of local populations**[**^21,^**](bookmark://_ENREF_21) [**^81^**](bookmark://_ENREF_81) - **Co-locate both sectors’ services to promote geographical proximity of PC and PH professionals and programs** [**^21,^**](bookmark://_ENREF_21) [**^81^**](bookmark://_ENREF_81) - Address health inequalities through ‘one-stop’ centers located in disadvantaged regions and organized around needs of local populations  1. **Coordinate clinical services with community services, whereby clinical services such as prevention, diagnosis, and treatment or rehabilitation are combined with services such as counselling, outreach, and social programs**[**^13,^**](bookmark://_ENREF_13) [**^21^**](bookmark://_ENREF_21) 2. Use combined data analytic resources of PH, Medicaid, and Medicare to inform development strategies that best deploy health promotion and care services to vulnerable populations in targeted geographic areas and decrease duplication efforts (see Data and IT Capabilities) 3. Provide counseling and educational services directed at personal risk behaviors, the management of certain health problems, the use of health services, etc. 4. Provide outreach and case management services to identify health needs of individuals and promote compliance with complex treatment programs 5. Provide social services that address socioeconomic drivers of health 6. Have PH work with Accountable Care Organizations (ACOs) 7. Facilitate partnership between public and private sectors for the delivery of integrated health services  - Identify challenges (e.g., elements of service delivery that are lagging) and assess whether greater private sector engagement could improve performance - Conduct private sector assessment to ensure accurate information about the scope of private sector service delivery  1. **Bring personnel to existing practice sites to provide individual-level support services to patients**[**^13,^**](bookmark://_ENREF_13) [**^21^**](bookmark://_ENREF_21)**^,^** [**^39^**](bookmark://_ENREF_39)  - PC sites can lease certain services from PH departments and vice versa - Organizations can hire or contract professionals with expertise or experience in providing a desired service - PC or PH sites bring in outside personnel to provide individual-level support services for patients  1. Coordinate school-based care with PH, e.g., childhood immunization, identification of family distress at early stages, mental health support 2. State health agencies and Medicaid offices should work together to improve medication adherence through partnership, including reviewing health and pharmacy data, outreach, and policies      1. **Coordinate community health assessments**[**^13,^**](bookmark://_ENREF_13) [**^21^**](bookmark://_ENREF_21) 2. For planning and developing health programs and services (for both PC and PH) 3. To ensure that health programs and services offered by PC and PH are responsive to community needs 4. To allow efficient allocation of limited health resources 5. Community health assessments should aggregate data from multiple sources: quantitative data from electronic health records (EHRs), administrative databases or surveys, and qualitative information from community meetings, interviews, and focus groups (and if the data is analyzed from multiple perspectives) 6. **Expand surveillance beyond infectious disease to include chronic disease^13^** 7. Coordinate health improvement campaigns 8. PC practices based on geography have the opportunity to contribute to health promotion campaigns that are led by PH and local authorities 9. PC practices can lend their waiting rooms and authority to campaign materials 10. **PH can work with clinical practices to develop tools that providers can use or resources that providers can refer to; and PH can collaborate with HC to develop customized and targeted programs for a region or population**^47^      1. Create sustainable system to conduct current analysis of available resources, analyze resource gaps, develop a resource improvement plan, and outline monitoring and evaluation efforts (see Quality Improvement) 2. **identify and reduce duplicative services, programs, and/or resources—refining and prioritizing learning and improvement needs related to multisectoral partnership^39^** 3. Identify methods and tools for gathering information to address multisectoral partnership learning needs 4. Tax-exempt hospitals can include PH laboratories in their needs assessment and implementation strategies 5. State health agencies should participate in identifying gaps in services and opportunities for linking adults to community resources through partnerships between PH and PC      1. **Formalize communication processes between PH and HC/PC that are timely, inclusive, transparent, and effective**[**^24,^**](bookmark://_ENREF_24) [**^77^**](bookmark://_ENREF_77)**^,^** [**^81^**](bookmark://_ENREF_81) 2. Mechanisms should account for one-to-many kind of relationship, and constant changes in HC structure and personnel 3. **Establish standard, direct communication channels and practices that efficiently transfer information across systems and organizational boundaries^39^** 4. **Facilitate mutual awareness and meaningful knowledge of one another, roles, responsibilities, services, and activities with an understanding of each other’s perspective^81^** 5. **Use frequent and multilayered communication to spread the word about a program's vision and message^96^** 6. **Use a neutral forum for controversial discussions^96^** 7. Create PH channels to educate clinicians taking their media preferences into account; promote greater awareness of resources      1. **Intensify focus at local, state, and national levels on ‘population health’ and improving health of communities^61^** 2. Establish monitoring systems to detect progress in achieving population health and health equity (see Data and IT Capabilities)  - Note: population-based statistics and data can be used, for example, to identify gaps in access to preventive services, access to healthy food options, health disparities, burden of chronic disease in particular areas - Monitoring systems should report data for populations experiencing health disparities and related social determinants of health - Should include data on indicators for all priority health concerns and related behavioral risk factors - **Focus on high-priority health measures and carefully selected intermediate factors (given limited resources) and demonstrate progress on set of key metrics^61^**  1. Data should be available at regular intervals and at the level of those working together 2. **Use population-based analytic tools, such as clinical epidemiology, risk assessment, and cost-effectiveness analysis, to enhance practice management, for example, by informing decisions about practice-site, service provision at each site, practice staffing patterns, the need for patient education programs, etc.**[**^13,^**](bookmark://_ENREF_13) [**^21^**](bookmark://_ENREF_21) 3. **Use and share population-based information (e.g., about prevalent health problems, health risks within the community, and preventive services for particular patient groups) to enhance clinical decision-making**[**^13,^**](bookmark://_ENREF_13) [**^21^**](bookmark://_ENREF_21)  - Use district health profiles (statistical report on the neighborhood and residents concerned with health and health determinants) (the Netherlands, UK) based on PC registries and health surveys by PH to promote integrated planning of health activities and PH and PC collaboration  1. Use population-based information to determine health resource availability in particular geographic regions and to understand health inequalities 2. **Use population-based strategies, such as community-wide screening, case finding, and outreach programs, to direct patients to medical care^13^**      1. Focus collaboration to address upstream drivers of health 2. **Strengthen health promotion and disease prevention through public education, advocacy, and initiatives targeted at improving community health (e.g., childhood obesity, HIV and preexposure prophylaxis (PrEP), hypertension, diabetes mellitus)^21^** 3. Focus on health problems with prominent environmental, social, and behavioral risk factors such as lead toxicity, tobacco use, and domestic violence 4. Focus on lifestyle and behavioral causes, e.g., targeted counselling and educational materials about personal behaviors such as smoking, sedentary lifestyle, or heavy drinking, and referred to appropriate community programs 5. Focus on patients’ social or physical environment (e.g., test a disease contact or assess potentially toxic worksites/homes)      1. Focus collaboration on health dispartities 2. Use practice lists to design health interventions, track health outcomes, and target specific high-risk patient populations 3. Use standardized demographic data in information systems to identify gaps and point toward best practices for eliminating disparities (e.g., monitor health outcomes to determine where clinical services are being delivered or to target outreach efforts and media campaigns)      1. Consider structures and resources that will enable partnerships to recognize and respond effectively to unforeseen events, changes in political landscapes and evolving fiscal environments (see Table 2: Emergency Preparedness and Response) 2. **Use emergencies as an opportunity to increase focus on PH**[**^48,^**](bookmark://_ENREF_48) [**^61^**](bookmark://_ENREF_61)      1. Engage in advocacy for policy change 2. Work with local, state, and national governments to adopt a Health in All Policies approach to ensure that governmental infrastructure and policies foster integration 3. Work with local, state, and national partners to improve functionality and utility of EHRs that support data infrastructure and interoperability between PC and PH, including community benefit organizations 4. Advocate for regulatory frameworks and economic incentives that increasingly ensure that PH and population health are critical to private sector healthcare efforts, as opposed to “orphaned efforts” 5. Advocate for healthy public policies and partnerships and networks to improve population health and reduce inequities 6. Advocate for health-related laws and regulations for disadvantaged groups to enhance health equity |
| Physical Infrastructure, Medical Supplies, Technologies, and Supply Chains | 1. Ensure availability of the physical infrastructure necessary to deliver quality PH and HC services 2. Update existing, and invest in new, secure and accessible health facilities to provide effective services with reliable water, sanitation and waste disposal/recycling, telecommunications connectivity, and a power supply, as well as transport systems that can connect patients to other care providers 3. Invest in laboratory infrastructure      1. Ensure availability and affordability of appropriate, safe, effective, high-quality medicines and other health products and technologies through transparent processes to improve health 2. Establish a transparent process to ensure equitable (with respect to availability and affordability) distribution of appropriate, safe, effective, high-quality medicines and other health products and technologies      1. Improve clinical and population telehealth and health data systems 2. When evidence demonstrates effectiveness, scale up digital health interventions from pilot schemes, including the integration of digital technologies into existing health systems’ infrastructures and regulation 3. Explore digital technologies that allow for collection of data from the home 4. Ensure coverage adequately remunerates for use of digital technologies (see Funding and Finance) |
| Quality Improvement | 1. **Develop a learning environment that integrates measures for improved quality management**[**^11,^**](bookmark://_ENREF_11) [**^48^**](bookmark://_ENREF_48) 2. **Build a collaborative culture at the operational level that enhances quality improvement^11^**      1. **Develop a program or process to manage change and achieve quality improvement in public health policies, programs, or infrastructure based on performance standards, measurements, and reports^96^** 2. **Continuously assess and improve the quality of integrated PH and PC health services (at the local, regional/state, and national levels)**[**^77,^**](bookmark://_ENREF_77) [**^81^**](bookmark://_ENREF_81) 3. **Use standardized shared-health-information system for collecting data and disseminating information (see Data and IT Capabilities)**[**^77,^**](bookmark://_ENREF_77) [**^81^**](bookmark://_ENREF_81) 4. **Establish and apply performance indicators and measures**[**^48,^**](bookmark://_ENREF_48) [**^96^**](bookmark://_ENREF_96) 5. Develop metrics that incentivize improvement in health (i.e., true performance) rather than improvement in documentation or workflow 6. Develop hospital metrics for population health 7. Include patients and families in improvement efforts (maternal child health) 8. Employ rigorous quality-improvement science 9. Develop novel methods for collaborative quality improvement (QI) (i.e., involving all partners) (details not given)      1. **Develop accreditation systems and performance standards that create incentive for partnerships^61^** 2. Create incentives and metrics with a focus on social determinants, health equity, and population health      1. **Document and report progress in meeting standards and targets, and sharing that information through feedback**[**^48,^**](bookmark://_ENREF_48) [**^96^**](bookmark://_ENREF_96) 2. Work with partners to support the evaluation of local integration projects to enable comparisons, demonstrate returns on investment, including social return, and support the development of strong business cases to implement future integrated approaches 3. **Establish regular reporting for accountability to key stakeholders^48^** 4. Disseminate lessons learned and use this knowledge to accelerate the scale-up of successful strategies to strengthen PC-oriented systems |
| Leadership and Workforce Development | 1. **Establish well-aligned multilevel leadership^24^** 2. Note: leadership should bridge disciplines, programs, and jurisdictions to reduce fragmentation and foster continuity; clarify roles and ensure accountability; develop and support appropriate incentives; and manage change 3. **Include formal systems leaders and collaborative champions (individuals who possess the necessary process-oriented skill to keep collaboration going) ^77^** 4. Allow PH and HC to jointly designate highly qualified and dedicated persons to manage the partnership and its programs 5. **Use leadership roles (e.g., distributed leadership)^11^, servant-leadership model in which the leader places the needs of others first and shared power**[**^48^**](http://0.0.0.48/) 6. **Select for transformative leadership qualities (i.e., visionary, dedicated) and skills, expertise, and experience**[**^48,^**](bookmark://_ENREF_48) [**^61^**](bookmark://_ENREF_61) 7. **Ensure continuity of leadership^61^**      1. Ensure leadership commitment to population health      1. Develop leadership training for PH to develop chief health strategists      1. Develop region-wide workforce planning to identify current and anticipated gaps in the workforce and workable shared models and strategies to attract workforce^39^ 2. **Ensure adequate quantity, competency levels, and distribution of a committed multidisciplinary PC and PH workforce^81^** that includes facility-, outreach-, and community-based health workers supported by effective management supervision and appropriate compensation 3. Divert health workers from redundant programs (requires reassessing the continuing relevance of existing health programs) 4. Offer avenues for salaried PH and PC physicians for collaborative projects (UK) 5. (Re)train and impart skills to the workforce for multisectoral, collaborative work 6. **Include new set of core competencies for noncommunicable disease care that includes patient-centered care, partnering and team-based care, quality improvement, information and communication technology, and PH perspectives^81^** 7. Define competencies and training required 8. Provide continuous professional development for collaboration 9. Establish formats for accreditation and appraisal 10. Drive change within undergraduate and graduate medical education to ensure future physicians are prepared for a more integrated system and population health-based roles within the private sector, where business, PH, and health equity increasingly align 11. Leverage existing tools for training in core competency and skills in assessment, planning, implementation, evaluation, advocacy, and partnership development 12. **PH can work with HC to support them in achieving prevention and quality improvement goals^47^** 13. Provide cross-system professional practice training or education, networking, and support activities**^39^** 14. State health departments and Medicaid can support joint training opportunities for PC and PH workforces 15. Provide training, including using and maintaining integrated health information systems 16. Provide training and technical support for those working in collaborative partnerships 17. **Develop training grants and teaching tools that train health professionals for more integrated practice; tools should focus on cultural outreach, health education, and nutrition (aimed at CDC/HRSA but more broadly applicable)**[**^81,^**](bookmark://_ENREF_81) [**^85^**](bookmark://_ENREF_85) 18. **HRSA and CDC should work with CMS to identify regulatory options for graduate medical education funding that give priority to provider training in PH and PC settings^85^** 19. **HRSA should create specific criteria or preferences related to curriculum development and clinical experiences that favor the integration of PH and PC ^85^**      1. **Utilize clinicians (e.g., physicians, nurses, nurse practitioners, physician assistants) in key roles in team-based care in collaboration between PH and PC** 2. Encourage full scope of practice as well as flexibility of scope of practice 3. Utilize pharmacists in the delivery of medication management therapy 4. **Utilize nurses in filling care coordinator role in collaborative clinics and integrated care models, and program facilitator role to support transfer of PH knowledge to PC (e.g., communicable or chronic diseases)^10^** 5. **Utilize nurse- or pharmacist-led immunization clinics in community-based settings^10^** 6. Use of secondments (i.e., temporary relocation to exchange experience or skills) (Canada)      1. **Recognize the need for and commit to developing a trained workforce that can create information systems and make them efficient for the end user (aimed at CDC/HRSA but broadly applicable)^85^**      1. **HRSA and CDC should create all possible linkages among PC training programs, its PH and preventive medicine training programs, and Epidemic Intelligence Service (EIS) programs^85^** 2. **HRSA and CDC should explore whether training component EIS and the strategic placement of assignees in state and local health departments offer additional opportunities to contribute to the integration of PH and PC by assisting community health programs^85^** |
| Data and IT Capabilities | 1. **Use a standardized, interoperable, shared information system for collecting data and disseminating information (see Quality Improvement)**[**^77,^**](bookmark://_ENREF_77) [**^81^**](bookmark://_ENREF_81) 2. Information system should include broad range of data systems such as EHRs, vital records such as electronic birth certificates, surveillance systems targeted at communicable diseases, antibiotic resistance, or behavioral risk factors, and disease-specific registries such as cancer, trauma, asthma, tuberculosis, and immunization 3. **Join efforts to undertake an inventory of existing health and healthcare databases and identify new data sets, creating from these a consolidated platform for sharing and displaying local population health data that can be used by communities (aimed at CDC/HRSA but broadly relevant)^85^** 4. Use geospatial platform when combining clinical and PH datasets 5. Leverage the EveryONE Project and data collection on social drivers of health in EHRs 6. Develop and maintain infrastructure for data analytics 7. Data can be utilized to produce reports on health and disease status of patients to understand needs of practice population and identify specific actions to address local health needs 8. **Develop nationwide, interoperable electronic health data from private and public HC providers and PH entities, including medical records, overview of critical risk factors, laboratory data and prescriptions, using national ID number (EU)^5^** 9. **Available to patients^5^** 10. **Can be used for surveillance^5^** 11. **Anonymized data without patient consent (opt out policy) or with patient consent can be used for secondary research^5^**      1. Ensure data liquidity and partnerships to mobilize and leverage data as data sharing needs to be both timely and granular for action 2. Automate reporting system for surveillance (e.g., Hamilton, Canada, for influenza-like illness), system integrated with EHR (not clinician dependent on initiation or workflow), and weekly summary reports automatically transmitted to PH      1. **Develop cross-system/sector oversight of information systems to ensure ongoing compatibility and interoperability^39^** 2. Create centralized or horizontal structure with independent body that is able to improve aspects of interoperability 3. Coordinate among geographic regions, sectors, institutions and systems 4. Establish well-defined competencies and responsibilities 5. Address data-blocking practices that deliberately restrict data access (see Governance and Legal)      1. Prioritize data sharing at the federal, state, and local level to achieve a learning health system inclusive of public health (aimed at health systems and electronic health data repositories but broadly applicable) 2. Examine how to best use the Affordable Care Act’s community benefits requirement for nonprofit hospitals by coordinating alignment of the data collection process      1. Modernize infrastructure and develop capacity to extend beyond PH and HC (development of a ‘community health record’) 2. Efficient and timely access to relevant multisector community health information depends on interoperability and the ability of different systems and organizations to easily exchange and use information 3. Provide other stakeholder training and resources to develop the necessary epidemiology, informatics, and technical expertise and resources to maintain community health record 4. Support time and training for using and maintaining EHR and data infrastructure 5. Support resources and infrastructure to address data-quality issues (e.g., completeness and timeliness of stored data) 6. Develop data streams to help underpin efforts by social services partners      1. Improve data availability and standards 2. Need greater PC and outpatient data (as opposed to hospital-based data more robust) 3. Use all existing data (e.g., health interview surveys) to create a ‘community health record’ 4. Integrate data on health and social services (e.g., hospital discharge planning, disability, financial support/benefits, long-term care), as well as socioeconomic, education, environmental, and behavioral data and measures at relevant temporal and geographic scales to understand health, document disparities, and design and target effective interventions 5. **Minimize burden of data collection (e.g., secondary use of EHR, central registries, national health records)^5^** 6. Data within EHR systems are frequently provided in unstructured formats, presenting challenges for its use in disease surveillance, although data structure varies according to the category of information, with information on sociodemographics, diagnosis, and prescriptions the most likely to be structured 7. Improve level of completeness of EHR data (e.g., sociodemographic and diagnosis data being the most likely to be ‘always’ completed, while other types of data are not); level of completeness is also related to data format, with categories of data that are most likely to be reported in structured format also most likely to be ‘always’ completed 8. Use definitions and standards for data collection and description of meta-data      1. Facilitate data linkage by personal identifier and/or regional identifiers (e.g., zip/post code) 2. Data linkage is more common for inpatient, cancer registry, prescription, health insurance, need also for PC, population health survey data, MH inpatient, and long-term care data 3. Link data between health and socioeconomic information to address equity      1. Develop data protection policies for issues such as data access, sharing, consent, cybersecurity, privacy, interoperability and inclusivity, unique personal health identifiers 2. **Change legislative environment to overcome limitations of data protection without compromising citizen privacy (look to Nordic countries for models of this legislation, e.g., Sweden)^5^**      1. **Use modern health information, communication technology, and digital technologies for health in ways that facilitate access to care and service delivery, improve effectiveness and efficiency, and promote accountability**[**^13,^**](bookmark://_ENREF_13) [**^21^**](bookmark://_ENREF_21) 2. Develop interoperability of eHealth applications |
| Multisectoral Partnerships and Public Engagement | 1. Foster a collaborative organizational culture that is maximally inclusive 2. Partners should demonstrate an ability to move toward a common goal and culture (and organizational readiness) of collaboration 3. Develop consensus norms, rules, and processes with input from all members of partnership 4. Obtain leadership buy-in to collaboration and support from organizational leaders to leverage additional funds to continue the work 5. **Achieve mutual respect and trust: value the work of other sector (and mutually supportive), avoid turf protection, seek to build trust, share power and influence**[**^48,^**](bookmark://_ENREF_48) [**^61^**](bookmark://_ENREF_61) 6. Understand and be prepared for the challenges of working with newly formed partnerships 7. Foster mutual learning (e.g., foster discussions and solutions around historic and current structural racism and biases that lead to inequity in the community; educate local government, private industries, and other entities about how social factors impact health) 8. Ensure collaboration reflects community served in terms of membership diversity 9. Use network science and analysis techniques to understand multistakeholder networks to inform the development of networks in terms of efficiency, effectiveness, sustainability      1. **Involve community (community-based service providers, governmental units, schools and educational institutions, businesses),^1,48^** **patients or consumers in integration efforts**[**^39^**](http://0.0.0.39/) 2. **Partnerships to improve community health should include HC and PH departments as core partners, but over time, should engage a broad ranges of parties from the private and public sectors^61^** 3. **Raise visibility of partnership in community^48^** 4. **Community-informed needs assessment drives health system integration activities**[**^39,^**](bookmark://_ENREF_39) [**^61^**](bookmark://_ENREF_61) 5. **Involve community and public collaborators who are most likely to maximize the value of services delivered to patients^39^** 6. Consider using an epidemiologist to help communities understand the health issues they face 7. **Provide technical support to community partnerships^11^** 8. Engage and activate citizenry to develop and sustain community-tailored health programs (e.g., in the creation of healthy environments, to address injury prevention, to address family planning) 9. **Develop two-way communication channel with community, using tools such as social marketing and purpose-designed apps^39^** 10. **Investigate and implement strategies or tools to better capture community and consumer feedback and/or to advance stakeholder involvement**^39^      1. **Launch community-wide projects that bring public, private, and non-profit sectors together with PH and PC to define problems, develop solutions, and prioritize actions for community health problems^21^** 2. In the US, typical sectors/stakeholders in collaboration are government PH structures, health delivery systems, communities, academia, state-level professional medical associations, and employers and businesses 3. Ensure community voices in priority setting, practice planning, and decision-making using community-health needs assessments, community boards, and patient and family advisory councils 4. Engage in cross-sectoral education and training or conducting cross-sectoral research 5. Enable shared information, by including listservs and communities of practice 6. Interventions/solutions can have a significant impact on people’s health and well-being by being integrated with other local services, e.g., in relation to social isolation, housing, and fuel poverty 7. Interventions for managing multimorbidity need to address health education, literacy levels, mental health, social support, improved living conditions 8. In maternal child health, engage full spectrum of [non-clinical] providers from other sectors from pre-pregnancy to infant care      1. Build, fund, and strengthen screening and referral for community programs      1. Increase research-practice partnerships |

**Supplement Table 2: Policy actions and considerations for the integration of public health and health care specific to emergency preparedness and response,** (note: bolded text signifies action/consideration from document supported by case-examples)

| **Domain** | **Policy Actions and Considerations** |
| --- | --- |
| Funding and Finance | 1. **Allow for more flexibility in routine and emergency program funding streams to enable entities across sectors to directly meet the needs for PH surge capacity during times of crisis^159^** 2. In response to evolving epidemiological challenges (inclusive of environmental threats and extreme weather events) 3. **Includes rapid research funding mechanism for just-in-time studies related to emergency preparedness and response^147^** 4. **To address the specific needs of vulnerable populations^159^** 5. Provide standing PH emergency repsonse fund and faster supplmental funding      1. Funders (e.g., federal and state agencies) should audit existing policies and funding mechanisms and realign to support PH and primary care (PC) integration 2. HRSA should expand investments into federally qualified health centers (FQHCs) that drive both telehealth and co-location with PH 3. HRSA should establish new PH and PC collaboration and capacity partner grant to foster development of local multisectoral coalitions (see Multisectoral Partnerships and Public Engagement) 4. Congress should provide sustainable investment to expand successful community-based models that include integrated PH and PC activities      1. Provide funding to establish and maintain healthcare coalitions (HCC), including funding for coalition staffing and assessing needs for and dissemination of lessons/benefits learned from it 2. The federal government should consider alternative frameworks for HCC administration to enable HCCs to pursue alternative review streams 3. Incentivize HC to further support HCCs (e.g., Medicare reimbursements could be adjusted for healthcare systems that contribute to HCCs) 4. Explore additional sources for funding for HCCs (e.g., providing tax incentives or insurance benefits for increased private-sector investments)      1. Provide continuous funding and promotion of transnational collaboration (EU)      1. **Ensure that health-system financing arrangements prioritize essential services and PC appropriately^159^** 2. Ensure sustainable funding for shared foundational capabilities and infrastructure (e.g., communication and IT infrastructure)      1. Ensure adequate and consistent funding levels for underfunded entities including PH, PC, and safety-net institutions 2. Take steps to diversify revenue streams and increase balance sheet strength to bolster financial positioning 3. Examine avenues for bolstering financial stability, and explore opportunities to “braid and blend” funding for health and social services 4. Extend reimbursement flexibilities for new delivery models and provide regulatory clarity for post-pandemic payment and operations 5. Leverage pandemic-era initiatives to stabilize provider finances to create new pathways encouraging providers to enter into alternative payment models (APMs) 6. Use APMs as a vehicle for scaling site-of-service flexibilities beyond COVID-19 7. Broaden the accessibility of value-based payment programs for all provider types, with a focus on embedding accountability for health equity into APM design 8. Leverage APMs as the vehicle for extending COVID-19 flexibilities for telehealth utilization and reimbursement 9. Incorporate the lens of health equity when designing APMs and evaluating COVID-19 10. Flexibility of extension 11. Invest in the upstream drivers of health, including the social determinants of health, to create more resilient communities with systems to support the full scope of health needs 12. Develop, test, and improve charge capture, accounting, and other financial systems to track resources and ensure adequate and timely reimbursement 13. Develop financial incentives to improve reporting of laboratory testing data during PH emergencies 14. For post-acute care: Explore opportunities for payment and coverage reforms for skilled nursing facilities (SNF) and long-term care      1. Leverage momentum from the pandemic to streamline costs by stopping wasteful services 2. Evaluate evidence on utilization trends during the pandemic to support the de-adoption of low-value health services |
| Governance and Legal | 1. Require/mandate collaboration across PH and HC to mobilize, coordinate, and direct medical and PH assets/entities 2. **Vertical and horizontal coordination/integration**[**^124,^**](bookmark://_ENREF_124) [**^159^**](bookmark://_ENREF_159) 3. **Coordination should incorporate scientific advice^130^** 4. CMS should require and hold accountable healthcare organizations (ACOs) to invest in community-based care and social services that facilitate integration of PH and PC and improve social drivers of health      1. **Develop clear governance structures demarcating the roles and responsibilities for PH and HC stakeholders both during and after emergencies^159^** 2. Clearly define, assign, and test responsibilities in all sectors, at all levels of government, and with all individuals and ensure each group’s integration 3. **Roles and responsibilities for all key responder organizations: various PH entities, HC entities, and emergency services^159^** 4. **Each entity and its staff/workforce should be educated regarding one another’s roles and responsibilities** (see Alignment of Core Functions)**^159^** 5. The role of HCCs as a collective and that of each of its discrete members need to be clearly defined and continuously evaluated as a requirement for continued funding (see Funding and Finance)      1. The federal government should consider alternative frameworks for HCC administration to ensure a better balance between public health and healthcare leadership      1. Work with federal regulators and state insurance commissioners to determine a minimum package of benefit adjustments for health plans during PH emergencies 2. Collaborate with regulators to define billing codes for activation during PH emergencies 3. Investigate administrative mechanisms for coordinating action across commercial payers 4. Strengthen local and regional governance for coordination of vital data and to openly share data (see Data and IT Capabilities)      1. Establish reporting requirements for communicable diseases and develop ‘bidirectional’ data sharing agreements among PH, HC (including long-term care), laboratories and government that extend beyond PH emergencies (see Data and IT Capabilities) 2. Incentivize required and voluntary data reporting/sharing 3. Focus reporting requirements on reducing data reporting burdens and that support needs for PH and HC core functions 4. Reporting requirements should align with federal strategies for improving data surveillance 5. **Identify and address issues concerning legal authority and liability barriers to effectively monitor, prevent, or respond to a PH emergency^159^** 6. Remove regulatory constraints on healthcare provider licensure and scope of practice during pandemics and other PH emergencies in order to support telehealth, mobilization, and deployment of healthcare workers across state lines (see Leadership and Workforce Development) 7. Strengthen PH protections 8. **Explore the relevant privacy issues and the protection of information in after action reports from use in legal proceedings or other punitive actions against practitioners and organizations as has been done for peer review data in other field (e.g., aviation, and occupational health)^147^** 9. Create and implement central European laboratory for the governance of the European network for the surveillance of communicable diseases |
| Alignment of Core Functions (that overlap between PH and HC) | 1. Identify a new backbone national entity that can support collaboration to achieve unified policy recommendations across PH and HC entities (EU) 2. Establish permanent infrastructure with autonomous management of its own budget and funds (EU) 3. Create and stabilize national reference centers in the field of high-impact infectious diseases (reference and guidance institution) (EU)      1. Build upon existing HCC or multisectoral regional coalitions and their strategic plans to support collaboration across PH and HC entities during public health emergencies 2. HCC should (continue to) lead regional HC preparedness and response coordination 3. **Coalitions should engage governmental and nongovernmental agencies to identify and secure resources needed to strengthen preparedness, in public-private partnership^97^** 4. Develop a system of regional collaborative projects. Each project would focus on 1 or 2 specific threats such as large-scale trauma, burns, or infection disease outbreaks 5. HCC should support PH agencies with situational awareness and IT tools already in use to identify vulnerable populations and other unique needs making them vulnerable during PH emergencies (e.g., have electricity dependent assistive devices) 6. HCC should plan and conduct system-wide exercises that incorporate hospitals, EMS, emergency management organizations, public health agencies, and addition HCC member participation 7. HCC should maintain the ability to communication across all of its members, HC organizations, PH, emergency management, skilled nursing facilities (SNF) and long-term care (LTC) facilities, and the pubic 8. HCC should have visibility into member resources and resource needs to meet community health needs during a PH emergency 9. HCC should develop process and procedures to rapidly acquire and disseminate information across its membership      1. **Sustain essential PH and HC core functions/services during a PH emergency** 2. **Maintain baseline levels of essential HC services during emergency (including mental health services)** 3. **Create flexible plans and management structures, enabling them to cope with rapidly evolving circumstances and changes in patient expectations and demands (e.g., reprioritization of clinical activities by health workers)^159^** 4. **The capacity of the system should be enhanced through deferral of nonessential primary and acute care services^130^** 5. **Flexible plans should guide health systems in allocating scarce resources and HC services, thereby ensuring the best outcomes for the highest number of patients^159^**      1. Expand existing services to meet emerging and urgent patient needs, including mental health services and connections to community-based services, including food banks and shelters (see Multisectoral Partnerships and Public Engagement)      1. Develop/evolve and support robust emergency preparedness programs that include collaboration and information between health systems and PH entities (with consideration to address both short-term and sustained PH emergencies) 2. Develop and maintain robust communication channels between PH and HC 3. Utilize medical operations coordination cells (MOCCs) to enable patient load balancing and reduce morbidity and mortality (MOCCs can be activated at the sub-state, state, and federal levels) 4. Operational policies for MOCCs should be set by stakeholders including state health department, emergency management, hospitals (and their service line experts) 5. HCC could perform MOCC functions with adequate support and ability to scale up to state or regional level depending on the scope of the PH emergency 6. **Develop, test, and improve decision-making and response capability using a formal integrated Incident Command System or Structure (ICS) at all response levels; identify specific components to improve response (see Quality Improvement)^159^** 7. State and local emergency management and incident command structures should consider formally integrating HCCs into the existing response infrastructure 8. Maintain ‘always on’ programs/systems and partnerships that can rapidly scale if needed (e.g., regular simulations and other cross-sector exercises); plans should be regularly and rigorously exercised (also see Leadership and Workforce Development) 9. **Designate assessment and treatment centers during high consequence infectious disease event e.g., Ebola; protocols established with designated facilities for patient transfer and logistics^159^** 10. Plan should include coordination of few triage/assessment centers and secondary assessment clinics (to the limit of capacity), establishing a few alternate levels of care sites (to the limit of capacity) (Canada) 11. Plan should not include a dedicated influenza hospital or a dedicated long term care surge facility; hospitals are responsible for planning their own “surge’ sites (Canada) 12. Plan should include standard criteria for diagnosing and assessing disease severity, for admission into hospitals and critical cares services, and for providing ventilator support (Canada) 13. Create reusable standards and protocols for PH emergencies and disaster medical responses 14. Include mental health and mental health providers as part of programs and collaboration 15. **Research included in emergency preparedness programs, with protocols to rapidly identify and procure investigational drugs and therapies^147^**      1. **Harmonize vertical PH and HC programs with horizontal health system-strengthening efforts prior to emergencies improves the provision of both baseline and emergency health services^159^** 2. **Collaboratively define protocols for crisis situations, including roles, responsibilities, and resources^159^** 3. **Routine updating of standardized protocols, definitions, and strategies for optimal clinical management^159^** 4. Identify and map existing resources and weaknesses across PH and HC entities’ capacities to determine priority needs      1. Monitoring health status/health risk assessment: Identify the hazards and vulnerabilities (e.g., community health assessment, populations at risk, high-hazard industries, physical structures of importance) that will form the basis of planning 2. Process to understand risks for the community, access and analyze information; facilitates informed planning and decision-making 3. Expand investments and partnerships to address the social determinants of health 4. Expand to environmental and ecological assessments 5. Integrate/coordinate systems to monitor, detect, and investigate potential hazards, particularly those that are environmental, radiological, toxic, or infectious 6. Integrate surveillance strategies and databases (see IT and Data Capabilities) 7. Collaborate contact tracing policies, teams, and tracing technologies (can be facilitated with technology) 8. Develop, test, and improve community mitigation strategies (e.g., isolation and quarantine, social distancing) and countermeasure distribution strategies when appropriate 9. Leverage and expand Laboratory Response Network (LRN) beyond bioterrorism 10. Increase laboratory capacity with partnerships and coordinate diagnostic testing strategies 11. Increase laboratory capacity with focus on ‘new’ infectious diseases, not relying on routine diagnostics 12. Create and implement central European laboratory for the development of research and prevention, diagnostic and therapeutic methods to combat infectious and/or toxicological agents (EU) 13. Maintain and improve the systems to test for potential hazards, particularly those that are environmental, radiological, toxic, or infectious 14. Use PH epidemiologists to link PH and hospitals for syndrome surveillance, communicable disease management, and PH emergency preparedness and response^178^ 15. Ensure that jurisdictional PH infection and prevention programs (including healthcare-associated infection programs) participate in developing infectious disease response plans      1. Develop and coordinate between PH and HC sustainable state and local vector-control programs      1. Address mental health and substance abuse gaps, bolster crisis resources, and incorporate mental health first-aid and long-term treatment into joint PH and HC disaster response and recovery strategies      1. Bolster laboratory capacity by developing formal relationships between PH laboratories at the state and local level with commercial labs, hospital and academic labs, and the CDC |
| Physical Infrastructure, Medical Supplies, Technologies, and Supply Chains | 1. Invest in remodeling the built environment of HC facilities to better support surge capacity 2. **Construction of new facilities, converting public venues to treatment facilities, reconfiguring existing medical facilities^130^** 3. Improve the capability to provide mass health services (e.g., vaccination) 4. Construct new facilities, convert public venues to treatment facilities, reconfigure existing medical facilities^130^ 5. Use pharmacies to deliver vaccinations      1. Ensure isolation and treatment areas that have communication and remote monitoring, decontamination areas, and waste storage/transport and incineration      1. **Plan for weather interruptions in critical infrastructure and transportation, such as back-up systems or contingencies to transfer patients to alternate care sites^159^**      1. **Leverage telehealth (electronic platforms, telephonic case management) for delivery of core functions of PH and HC^130^** 2. Collaborate with providers and regulators to develop sector-wide standards for care quality and clinical appropriateness of virtual health services 3. Expand telehealth capabilities to include more effective linkages with urgent care, radiology, laboratory, and pharmacy 4. Dedicate resources to addressing potential inequities in patient access and the quality of virtual care 5. Improve telehealth capabilities in SNF, long-term care and home care sectors 6. Investigate care delivery demonstration models, and provide support for rural infrastructure investments in telehealth      1. Invoke a central/regional coordinator to manage an integrated supply chain for medical countermeasures, equipment, staff, and other critical resources during public health emergencies (see Alignment of Core Functions)      1. **Identify and manage critical resources for PH and HC emergency response and practice and improve the ability to deliver these resources throughout the supply chain^159^** 2. **Reevaluate vendor selection and GPO contracts, and explore opportunities for regional collaboration^130^** 3. **Develop a regulatory mechanism for tracking and reporting inventory across systems and stakeholders (including platform to monitor supply chains)^130^** 4. **Increase funding for the Strategic National Stockpile and update protocols for resource allocation^130^** 5. **Develop and sustain robust public-private partnerships for effective medical countermeasures development and production^130^** 6. **Utilize informed and fair principles for rational use of products^130^** |
| Quality Improvement | 1. **Make commitment to and transparency of continuous quality improvement (that promotes excellence and garners the trust of the community)^159^** 2. **Develop robust measurement strategy with structural and process, and outcome metrics to support emergency response^147^** 3. Use a strategic planning process that includes national, regional and institutional leaders to specify goals and metrics of readiness for hospitals, HC, PH, regional coalitions 4. **Raising standards and expectations regarding the quality of information^147^ reported in after action reports by defining its essential core elements** 5. **Should include individual and population outcomes^147^** 6. **Should include short term metrics (days to weeks) and longer-term metrics (months)^147^** 7. **Establish an independent review panel with a standardized after-action reporting process with aims of reducing bias and increasing the utility of after-action reports produced following PH emergency responses^147^** 8. **National repository of after-action reports or reports analyzing after action reports that is accessible to support dissemination of key findings, lessons learned, and best practices^147^** 9. **Monitoring and evaluation around how and in what circumstances an ICS should be implemented^147^**      1. Rethink arduous process of data capture, implementation of novel measures and removal of measures that are no longer useful 2. Develop new metrics and incentives for achieving equity in PH and HC 3. Create and implement local, state, and regional metrics for health status and assessment of vulnerabilities using sensible geographic demarcations      1. Explore opportunities to align incentives to address disparities in care quality and outcomes 2. Expansion of payment incentives directed at HC delivery mitigating population health vulnerabilities caused by social and environmental determinants      1. The Patient-Centered Outcomes Research Institute, CMS Innovation Center, and Agency for Healthcare Research and Quality should drive innovation in PH and PC models to improve community- and patient centered care using research and sharing of best practices      1. Develop and implement an accreditation or certification program for HC to encourage and recognize them for progress toward specific, measurable, regional objectives in strengthening collaboration with PH and their readiness for disasters |
| Leadership and Workforce Development | 1. Intentionally train, recruit, and develop PH and HC leaders 2. Strengthen leadership for health equity 3. **Implement skilled, flexible leadership and clear command structures^159^** 4. Develop joint leadership structure      1. Develop and maintain a PH and HC workforce that has the skills and capabilities to perform optimally in a PH emergency 2. Evaluate opportunities to standardize HC workforce regulations for emergencies 3. Remove regulatory constraints on healthcare provider licensure and scope of practice during PH emergencies in order to support telehealth, mobilization, and deployment of healthcare workers across state lines 4. **Fund and develop programs and resources to support the ongoing professional development of the incumbent and pipeline workforce to meet the current population health needs^130^** 5. Fund interprofessional education and training of the PH and PC workforce—medicine, nursing and PH—to establish increased understanding and a precedent for team-based communication and collaboration. 6. Academic programs to create and sustain a pipeline for the PH and PC workforce with the knowledge, skills, and abilities to serve the needs of US communities during future infectious disease outbreaks should be expanded to accommodate physicians and nurses who wish to pursue a master’s degree in PH. 7. ‘Mystery’ patient drills and other exercises to help identify areas needed for training 8. Explore updates to continuing medical education 9. **Train and staff for disaster mental health**[**^130,^**](bookmark://_ENREF_130) [**^159^**](bookmark://_ENREF_159) 10. **Develop robust communication plans to update, and train staff^159^** 11. Develop programs and train PH and HC staff in communications skills; communications training should include how to combat myths and disinformation, training for use of social media and other modes of communication 12. Engage partnerships among HCCs to identify needs and mechanisms for training      1. **Develop strategies to rapidly expand workforce and flexible staffing models; can involve (temporary, mandatory) reallocation of PH and HC professionals**[**^130,^**](bookmark://_ENREF_130) [**^159^**](bookmark://_ENREF_159) 2. HC providers who are willing to be redeployed should be adequately compensated and protected 3. **Create voluntary or involuntary reserve of adequately trained personnel^159^** (e.g., volunteers may be from the Medical Reserve Corps or registrants with the Emergency System for Advance Registration of Volunteer Health Professions) 4. Collaboration with partner organizations to increase response capacity 5. Invest in robust PC workforce (PC and community health centers can play an important role in HC and community resilience and mitigating burden on hospitals through integration in medical home model) 6. Allow for flexibility of scope of practice (e.g., pharmacists to administer vaccinations, medical assistant and office personnel supervised by PC clinicians to assist in PH contact tracing)      1. Support the retention and recruitment of diverse PH and HC professionals and leaders who are representative of the community they serve, with updated mechanisms to ensure appropriate compensation and recognition 2. Bolster protections for PH and HC staff 3. Reaffirm a commitment to investing in workplace wellness and culture of workplace safety, including new and preexisting strategies to reduce clinician burnout 4. **Ensure mental health support for workforce^130^**      1. Fortify state/regional healthcare provider data such that the Office of the Assistant Secretary for Preparedness and Response can establish a unified national registry of medical practices and licensed healthcare providers to facilitate timely and relevant communication from federal authorities to healthcare providers and provide an important preparedness mechanism that can be accessed during a PH emergency. |
| Data and IT Capabilities | 1. Develop data infrastructure needs to severe multiple purposes: for example, coordination, forecasting (for program planning, allocation of resources), surveillance (disease surveillance, pathogen surveillance), and quality and research      1. Improve multi-stakeholder and multidisciplinary data infrastructure by engaging data providers and decision-makers from the PH, HC, research, and technology entities, as well as the governance of all included nations and regions 2. Develop effective public-private partnerships 3. Eliminate duplication and administrative burden of data sharing and reporting 4. Leverage membership and claims data and improve data sharing capabilities with PH to support disease surveillance and population health monitoring 5. Include data from other stakeholders like LTC facilities and other congregate settings where testing is done, or syndromic data can be collected      1. Public Health Data Systems Task Force should consider defining a core dataset for PH emergencies developing additional standards for data collection, developing a plan for implementing those standards and linking them to funding mechanisms (see Funding and Finance, Data and IT Capabilities) 2. Core data set should include information for PH emergency surveillance and response, including demographic information, electronic laboratory data, travel health data, genomic sequencing data, and electronic vital records data 3. Modernize core pieces of PH data infrastructure (e.g., National Notifiable Diseases Surveillance System, vital records system)      1. Ensure that interoperable digital infrastructure is made available to enable facile information sharing between health systems (and their EHRs) and PH entities (as well as across health systems and their EHRs) 2. Greater investment into robust and interoperable (and secure) health IT infrastructure, consistent architecture and standardized components, equitably distributed IT infrastructure 3. The National Academy of Science, Engineering, and Medicine should seek funding for a study to propose a process for developing a national-, state-, and local-level data infrastructure for sharing information across PH and HC 4. Congress should direct HHS to ask National Academy of Medicine to propose a design for a national interoperable data platform to improve access to health and other relevant data during ongoing PH emergencies 5. IT infrastructure to include laboratory (PH, clinical, commercial) 6. Establish national standards to enhance PH and HC IT system interoperability 7. Data standards and terminologies to support clinical research are available now; they should be leveraged, aligned with other standards as appropriate, and widely adopted for all necessary data inputs into the system 8. Data transmitted across local, state, and regional PH departments, schools, and outpatient delivery entities as well as short- and long-term institutional living facilities 9. Review of performance of ‘meaningful use’ should include health system integration with PH services 10. **Create efficient data collection with minimal disruption to delivery of services^147^** 11. Investment into national health record (or centralized databases) as mechanisms for responsive, accountable, and equitable resource allocation (non-US)      1. Modernize syndromic surveillance system 2. CDC should establish an integrated infectious disease surveillance system that would strengthen and pull together efforts conducted by multiple sectors, agencies, and data systems 3. Include novel signals from data sources such as social media, electronic health records, eHealth platforms, and crowdsourcing 4. Develop more nimble system that allows development, collection, calculation, and analysis of new metrics in real time 5. Utilize advanced AI powered processing to enhance its learning capabilities 6. Develop integrated system(s) to predict emergence of zoonotic disease by epidemiologic modeling of human and animal interfaces, enhancing wildlife and bio surveillance methods 7. ‘Health information systems’ should be developed and implemented to address the ‘infodemic’ by helping to identify sources of misinformation      1. Improve upon translation of data (see Quality Improvement) 2. Define key elements for information sharing (e.g., bed capacity, medical supplies) 3. Set national standards to ensure that health data is routinely disaggregated by race, ethnicity, and other key sociodemographic characteristics to the community level (as appropriate to ensure anonymity) to identify disproportionate health impacts and outcomes |
| Multisectoral Partnerships and Public Engagement | 1. Establish and maintain regional and/or state-level backbone entities or multisectoral coalition (e.g., HCC) that can be leveraged during crises for shared action (see Alignment of Core Functions) 2. Plan with communities, not for them, and empower their involvement with resources 3. Provide resources and technical assistance to communities to enhance equity and resilience before, during, and after a public health emergency 4. **Develop new and enhance existing partnerships both within the health system and among other sectors, enhance resilience by improving emergency response time and access to financial, intellectual, and human resources (e.g., regional, institutional, local governmental agencies, emergency medical services, social and PH sectors, employers, business sector, community-based organizations)^159^** 5. Cultivate relationships with non-traditional partners including employers, the business sector, and technology 6. **Foster partnerships with researchers and industry to support clinical trials enrollment and research on preparedness, response and recovery phases of PH emergency^147^**      1. Address public’s erosion of confidence in PH and HC with strong partnership with community and robust communication      1. Develop strong partnerships with the community 2. Develop understanding of community priorities and values 3. Identify the capacity and limits of community health resilience based on multiple community metrics 4. **Engage and train community-based partners serving at-risk populations to improve community preparedness efforts (accompanied by targeted monitoring and outcome evaluation to improve evidence base for engagement and training strategies)^147^** 5. Educate, engage, and mobilize the public to be full and active participants in PH emergency preparedness 6. Focus on health equity, community resilience, and sociopolitical factors that affect health 7. Ensure two-way channels for public information and communication; PH and HC should serve as a trusted source of accurate, timely PH education and information 8. Develop, practice, and improve the capability to rapidly provide accurate and credible information to the public in culturally appropriate ways 9. Enlist and collaborate with people who ‘look like’ who you are trying to communicate with 10. Identify and prevent the spread of misinformation 11. Use social media in coordinated fashion (when, what, and to whom) 12. **Use SMS text messaging and other electronic messaging channels^147^** 13. **Bolster emergency risk communication strategies (i.e., real time exchange of information, advice, and opinions among decision-makers, experts, and general public) providing up-to-the-minute information for the community^130^** 14. **Place emergency risk communication in (national) leadership structure, create organizational proximity of communication practitioners to national response leadership, develop laws, regulations, policies and frameworks in support of emergency risk communication, use trainings and exercises as mechanisms for testing the effectiveness of the system^165^** 15. **Improve capability to obtain feedback from public/community^130^** |

**Supplement Table 3: Selected case examples of public health and health care integration reporting objective measures of success**

| **Author, Year**  **Country** | **Category**  **Subcategory** | **Description** | **Results** | **Themes** |
| --- | --- | --- | --- | --- |
| Cramer, 2020^6^  2020  US | PH-HC    General | Investigated whether individuals residing in communities with stronger collaboration between nonprofit hospitals and local public health departments (LHDs) reported healthier behaviors. | Stronger collaboration between nonprofit hospitals and LHDs was associated with not smoking (odds ratio, OR 1.32, 95% CI 1.11 to 1.58), eating vegetables daily (OR 1.29; 95% CI 1.06 to 1.57), and vigorous exercise (OR 1.17; 95% CI 1.05 to 1.30). The presence of higher social capital also strengthened the relationships between LHD-hospital collaborations and wearing a seatbelt (p for interaction = 0.01) and general exercise (p for interaction = 0.03). | Unable to assess |
| Fukada, 2020^16^  US | PH-HC    Infectious disease (HIV, viral hepatitis, STI) surveillance | Massachusetts Department of Public Health worked to integrate its HIV, viral hepatitis, sexually transmitted infection (STI), and tuberculosis response through policies that mandated contracted organizations to submit specimens for testing to the Massachusetts State Public Health Laboratory; co-test blood specimens for HIV, hepatitis C virus (HCV), and syphilis; integrate HIV, viral hepatitis, and STI disease surveillance and case management in a single data system; and implement an integrated infectious disease drug assistance program. | HIV tests increased by 106%. New HIV diagnoses decreased from 191 to 160. HCV infection increased by 205%.  New HCV diagnoses increased from 687 to 4971. HCV tests with positive results increased from 6% to 14% when HCV co-testing was initiated.  Syphilis tests increased by 61%. New syphilis diagnoses increased from 531 to 1539. Syphilis tests with positive results increased from 3% to 6% if co-testing was added.  Linkage-to-care rates increased from 69% to 80% among persons newly diagnosed with HIV infection, and 7% to 48% among persons newly diagnosed with HCV infection. Referral to STD treatment for persons diagnosed with syphilis increased from 68% to 80%. | Funding and finance, governance and legal, physical infrastructure, leadership and workforce development, multisectoral partnership and public engagement |
| Livingston, 2020^14^  US | PH-HC    Tobacco | Creation and implementation of a novel incentive metric for Oregon’s Medicaid delivery organizations for smoking cessation. Oregon Health Authority worked to connect the statewide network of Tobacco Prevention and Education Program in all counties and tribes with their regional Coordinated Care Organizations (CCOs) to replicate the comprehensive scope of tobacco use prevention and cessation activities at the local level. | Thirteen of 15 CCOs demonstrated a reduction in smoking prevalence, with the statewide prevalence decreasing from 29.3% to 26.6%. | Governance and legal, quality improvement, leadership and workforce development, multisectoral partnership and public engagement |
| Ross, 2020^12^  US | PH-HC    Pharmacy (MTM) | To explore pharmacists’ role in medication therapy management (MTM) to improve cardiovascular disease outcomes in residents of regions like the Mississippi Delta that have high rates of poverty, health disparities, and poor health outcomes. | Significant mean reductions occurred in the following metrics: systolic blood pressure (SBP) (7.1 mm Hg), diastolic blood pressure (DBP) (6.3 mm Hg), LDL cholesterol (24.9 mg/dL), triglycerides (45.5 mg/dL), total cholesterol (37.7 mg/dL), and HbA1c (1.6% [baseline >=6%] and 1.9% [baseline >=9%]). For patients with diabetes, significant reductions in HbA1c were demonstrated. Patients with the highest risk for diabetes complications (baseline HbA1c >9%) experienced a 1.9% reduction in HbA1c (for a 17.1% relative reduction). Similarly, SBP and DBP (analyzed separately) were significantly lower following receipt of MTM services. | Leadership and workforce development |
| Thompson, 2020^9^  US | PH-HC    Pharmacy (MTM) | To evaluate changes resulting from pharmacist-delivered MTM services in participant knowledge and health beliefs about hypertension, use of blood pressure self-management tools (logs and monitors), and medication adherence barriers. | We observed improvements in self-reported use of self-management tools: log to track blood pressure readings (P < .001), log to track antihypertensive medication use (P=0.045), self-measuring blood pressure monitors (P < .001).  Decrease in proportion of participants reporting difficulty: remembering their medication dosage (decreased by 50%), remembering to take medications (decreased by 60%). | Leadership and workforce development |
| Carlton, 2018^37^  US | PH-HC    Community health needs assessments | To examine the association between hospital-local health department collaboration on community health needs assessments and hospital investment in community health. | Data from 439 local health departments in the United States. The regression models showed that each increase in the number of implementation planning activities reported by an LHD was associated with a statistically significant increase in the proportion of hospitals’ total budget spent on community  health improvement initiatives by 0.09. | Alignment of core functions |
| Main, 2018^36^  US | PH-HC    Maternal health | California Maternal Quality Care Collaborative (CMCC) was formed as a public-private partnership to lead maternal quality improvement activities. Key steps undertaken included linking public health surveillance to actions, mobilizing a broad range of public and private partners, developing a rapid-cycle Maternal Data Center to support and sustain quality improvement initiatives, and implementing a series of data-driven, large-scale quality improvement projects. | US maternal mortality worsened in the 2010s; in contrast, by 2013, California’s rate had been cut in half to a 3-year average of 7.0 maternal deaths per 100,000 live births (comparable to the average rate in Western Europe).  Reduction of severe maternal morbidity (SMM) among hemorrhage patients in the California Maternal Quality Care Collaborative (CMQCC) for obstetric hemorrhage, by hospital group: hospitals in CMQCC hemorrhage collaborative (n = 99): 20.8% (p<0:0001) (hospitals in CMQCC; with prior hemorrhage collaborative experience (n = 25): 28.6% (p<0:0001)); hospitals not in CMQCC hemorrhage collaborative and with no prior hemorrhage collaborative experience (n = 48): 1.2% (p=0.7713) | Alignment of core functions, quality improvement, data and IT capabilities, multisectoral partnerships and public engagement |
| Nuffer, 2018^35^  US | PH-HC    Pharmacy (MTM) | Describes a partnership between a state public health department, a regional care collaborative organization (RCCO), and a school of pharmacy to establish pharmacy-delivered care services for patients in rural Colorado, including a centralized, public health pharmacist to help coordinate these efforts. | Seven of 16 patients (44%) were successfully changed from Crestor to the generic therapy, representing an estimated cost savings of $6,500/year, or $929 per patient per year.  The statin initiation initiative identified 190 patients across the sites eligible for therapy, and 51 (26.8%) were successfully started on statin therapy (projected to prevent one heart attack over the next 5 years). Pneumococcal initiative successfully immunized 32 of 45 potential candidates (71% success rate) in the three communities. | Funding and finance, alignment of core functions, data and IT capabilities |
| Gosling, 2016^52^  UK | PH-HC    Primary care,  Tobacco | SmokeFree Liverpool is a system-wide partnership whose strategy was to achieve local smoke-free legislation while encouraging local workplaces to become voluntarily smoke-free and establishing a comprehensive smoking cessation service. Primary care was incentivized to offer smoking cessation advice and referral to the specialist cessation service. | Between 2005 and 2012, smoking prevalence fell from 35% to 24.5%. At its peak, 50% of referrals to the smoking cessation service came from primary care. | Alignment of core functions, quality improvement, leadership and workforce development, data and IT capabilities |
| Terranova, 2016^46^  US | PH-HC    Infectious disease (HIV, viral hepatitis, STI) | Partnership between the New York City Department of Health and Mental Hygiene and six federally qualified health centers (FQHCs) to make screening for HIV and HCV routine and increase adherence to gonorrhea treatment guidelines through education, electronic health record modification, and progress tracking. | After 1 year, among the 14 clinics, 12 documented improvements in their HIV offer rates and 11 documented improvements in their HIV screening rates. On average, the percentage of patients who were offered HIV screening increased from 26% at baseline to 56% at follow-up. On average, the FQHC screening rates for HIV increased from 25% at baseline to 38% at follow-up. | Alignment of core functions, leadership and workforce development, data and IT capabilities |
| Bruckner, 2014^66^  US | PH-HC    Chronic disease, cancer | Review of community health needs assessment and subsequent work conducted by Macon County Public Health and healthcare partners for three health priorities: reduction in the incidence of preventable chronic diseases related to obesity; improved access to care through recruitment and retention of additional primary care physicians and dental practitioners; and reduction in the incidence and mortality rates of breast, colon, and lung cancer through prevention and early intervention. | Five-year diabetes mortality rates fell to 16.8 per 100,000 population in 2008–2012 from 23.0 per 100,000 population in 2003–2007, and 71% of the patients who completed MCPH’s diabetes self-management training program reduced their glycosylated hemoglobin level to 6.5% or lower. In addition, participants in Angel Medical Center’s Lighten Up 4 Life program collectively lost more than 4,775 pounds. | Alignment of core functions |
| Kempe, 2014^63^  2014  US | PH-HC    Primary care, pediatric vaccination | Public-private collaboration for delivering influenza immunization to children.  Four pediatric and four family medicine (FM) practices randomized to intervention (joint community clinics and public health department nurses aiding in delivery at practices) or control involving usual care without PHD. | Immunization rates increased by 9.2% in intervention and 3.2% in control (p<.0001), with significant increases in both pediatric and FM practices. Largest increases among school-aged and adolescent children (p<.0001 for both). Differences for 6-month-old to 5-year-old children and  for children with high-risk conditions did not reach statistical significance. | Alignment of core function |
| Villanueva, 2013^67^  US | PH-HC    Cancer screening (CRC) | Describes the planning and development of a multisite, multiorganizational collaborative screening program in Baltimore, Maryland, an urban setting, and the implementation and operation of the program, including fiscal, organizational, programmatic, and clinical challenges and successes, as well as screening outcomes. | The program resulted in 709 colonoscopies screening cycles performed among 696 people and detect three cancers during the 38 months of screening. | Funding and finance, governance and legal, multisectoral partnership and public engagement |
| Dukers-Muijrers, 2012^89^  Netherlands | PH-HC    Infectious disease (STIs) | To close the gap in sexual health care by implementing and evaluating a policy change regarding the combination of public health and hospital care in an innovative, integrated STI/HIV care structure serving male and female HIV patients in the Netherlands. | Pre-intervention, 43% of the patients wanted to discuss sexual health (51% MSM; 30% heterosexuals). Of these patients, 12% to 35% reported regular coverage, and up to 25% never discussed sexual health topics at their HIV care visits. Of the patients, 24% used our intervention. Usage was higher among patients who previously expressed a need to discuss sexual health. Most patients who used the integrated services were new users of public health services. STIs were detected in 13% of MSM and in none of the heterosexuals. | Alignment of core functions |
| Klompas, 2012^84^  US | PH-HC    IT | Describes a model EMR-based public health surveillance platform called Electronic Medical Record Support for Public Health (ESP). The ESP platform provides live, automated surveillance for notifiable diseases, influenza-like illness, and diabetes prevalence, care, and complications. Results are automatically transmitted to state health departments. | Over 5 years, over 12,500 case reports have been submitted by ESP installations to the Massachusetts and Ohio state health departments. | Data and IT capabilities |
| Herwehe, 2011^86^  US | PH-HC    HIV/AIDS | Describes the Louisiana Public Health Information Exchange (LaPHIE), a secure bidirectional public health information exchange linking statewide public health surveillance data with electronic medical record data. Alerts medical providers when individuals with HIV/AIDS who have not received HIV care for >12 months are seen at any ambulatory or inpatient facility in an integrated delivery network. | Between 2/1/2009 and 1/31/2011, 488 alerts identified 345 HIV-positive patients. 76% of the patients were aware of their HIV status but had not received care for >12 months. The majority (82%) followed up with HIV care within the study period, with 82% receiving at least one CD4 count during the 18-month follow-up study period and 62% having at least one HIV specialty visit. | Alignment of core functions, data and IT capabilities |
| Freeman, 2010^93^  US | PH-HC    Mental health | In partnership with Seattle’s Aging and Disability Services and Senior Services of Seattle/King County, the Program to Encourage Active, Rewarding Lives for Seniors (PEARLS), a randomized control trial in Seattle/King County, aimed to reduce minor depression and resulting disability among older adults by teaching them depression-management techniques. | Patients who received the intervention were significantly more likely to have a 50% or more reduction in depressive symptoms, remission from depression, and have improved functional and emotional well-being. No difference seen in health care use. | Alignment of core functions, quality improvement, leadership and workforce capacity |
| Iliffe, 2003^105^  US | PH-HC    Primary care | The article reviews the experience of implementing Community Oriented Primary Care (COPC) methods. Included case study: Tri-County Family Medicine nonprofit practice, rural western New York State Migrant health outreach clinics. | Improved uptake of preventive services, from 58% to 78% of population by 2 years. | Multisectoral partnership and public engagement |
| ASPR, 2022^145^  US | Emergency preparedness    Medical operations coordination cells (MOCC) | The Washington Medical Coordination Center works to prevent strain on hospitals by load-balancing acutely ill patients across the state (COVID-19 as well as other acute illnesses) for hospitals impacted by pandemic-related surges and staffing challenges. Center utilizes statewide operational agreements and bed surveillance of the health system to ensure access to care regardless of from which hospital a patient may originate. | As of early January 2022, the WMCC had received over 3,800 hospital requests for assistance and had completed over nearly 2,000 load-balancing patient placements. ​ | Alignment of core functions, multisector partnership and public engagement |
| Lim, 2022^113^  Australia | Emergency preparedness    COVID | COVID Positive Pathway, a collaborative model of care involving the Victorian public health unit, hospital services, primary care, community organizations, and the North Western Melbourne Primary Health Network to support people with coronavirus disease 2019 (COVID-19) isolating at home. | About 80% of participants could be adequately supported by primary care and community organizations, allowing hospital services to be reserved for people with more severe illness or with risk factors for disease progression. | Alignment of core functions |
| Rubin, 2014^173^  US | Emergency preparedness    H1N1 | Describes how local health departments can most effectively develop and maintain relationships with community pharmacies and pharmacists that will allow for a more coordinated and resourceful public health response to emergencies, specifically to pandemic influenza outbreaks. | Palm Beach 2009 H1N1 Response: More than 200,000 Flu Ready cards were distributed at 250 pharmacies between September 2009 and March 2010. By identifying one contact at each pharmacy retailer, Palm Beach County Health Department was able to ship approximately 40,000 doses of the 2009 H1N1 vaccine, about 12% of the county’s allocation, to hundreds of instore health clinics and community pharmacies. | Alignment of core functions, leadership and workforce development, data and IT capabilities, multisectoral partnership and public engagement |
| Wynn, 2012^175^  Canada | Emergency preparedness    H1N1 | Collaboration between Ontario public health unit and its primary care providers during H1N1 influenza pandemic (family health team [FHT]) to facilitate response during public health emergencies, in partnership with public health. | FHTs were able to cover 55% of patients within region. Reports on patient and physician satisfaction with FHT management and ability of ongoing surveillance to trigger opening of Flu Assessment Centers. | Alignment of core functions |
